# Supplementary material for: A recent update on the morphological classification of intraductal papillary neoplasm of the bile duct: Correlation with postoperative prognosis and pathological features
Source: PLoS One. 2025 May 28;20(5):e0325081. doi: 10.1371/journal.pone.0325081 (PMC12118913; doi:10.1371/journal.pone.0325081)
Supplement: S1 Table — Abbreviations: CIS, carcinoma in situ; IPNB, intraductal papillary neoplasm of the bile duct. (DOCX) [file pone.0325081.s001.docx]

**S1 Table. Patient demographics according to pathological subtype**

| **Variables** | **Type 1**  (n=10) | **Type 2**  (n=22) | ***P value*** |
| --- | --- | --- | --- |
| **Age (y.o.)** | 73 [66–75] | 75 [72–78] | *0.159* |
| **Gender (Male / Female)** | 7 (70) / 3 (30) | 12 (55) / 10 (45) | *0.335* |
| **Preoperative biliary drainage (%)** | 1 (10) | 2 (9) | *0.690* |
| **Symptoms (%)** |  |  |  |
| **Cholangitis** | 2 (20) | 5 (23) | *0.624* |
| **Jaundice** | 1 (10) | 5 (23) | *0.373* |
| **Fever** | 1 (10) | 2 (9) | *0.690* |
| **Abdominal pain** | 1 (10) | 3 (14) | *0.632* |
| **Tumor findings by preoperative image study (%)** |  |  |  |
| **Morphologic classification: Branched / Main duct / Mixed** | 6 (60) / 2 (20) / 2 (20) | 4 (18) / 14 (64) / 4 (18) | ***0.039*** |
| **Lesion extent: Intrahepatic / Extrahepatic / Both** | 7 (70) / 2 (20) / 1 (10) | 6 (27) / 9 (41) / 7 (32) | *0.072* |
| **Cyst** | 7 (70) | 8 (36) | *0.083* |
| **Bile duct dilation without obstruction** | 2 (20) | 7 (32) | *0.405* |
| **Bile duct wall thickness** | 5 (50) | 15 (68) | *0.438* |
| **Mural nodule** | 9 (90) | 18 (82) | *0.494* |
| **Enhanced mural nodule** | 7 (78) | 12 (67) | *0.450* |
| **Lymph node swelling by image study** | 2 (20) | 1 (5) | *0.224* |
| **Pathological findings (%)** |  |  |  |
| **Low grade Intraepithelial neoplasia** | 6 (60) | 1 (5) | ***0.001*** |
| **High grade intraepithelial neoplasia (equivalent to CIS)** | 1 (10) | 3 (14) | *0.632* |
| **Invasive carcinoma** | 3 (30) | 18 (82) | ***0.007*** |
| **Invasion depth: M / FM / SS / SE or SI** | 3 (100) / 0 (0) / 0 (0) / 0 (0) | 5 (28) / 4 (22) / 2 (11) / 7 (39) | *0.128* |
| **Superficial extension** | 0 (0) | 10 (56) | *0.124* |
| **Lymph node metastasis** | 0 (0) | 3 (17) | *0.614* |
| **Residual tumor of R1 or 2** | 0 (0) | 7 (39) | *0.274* |
| **Mucin secretion (%)** | 8 (80) | 10 (46) | *0.073* |

Abbreviations: CIS, carcinoma in situ; IPNB, intraductal papillary neoplasm of the bile duct
