# Supplementary figures and images for: A recent update on the morphological classification of intraductal papillary neoplasm of the bile duct: Correlation with postoperative prognosis and pathological features
Source: PLoS One. 2025 May 28;20(5):e0325081. doi: 10.1371/journal.pone.0325081 (PMC12118913; doi:10.1371/journal.pone.0325081)

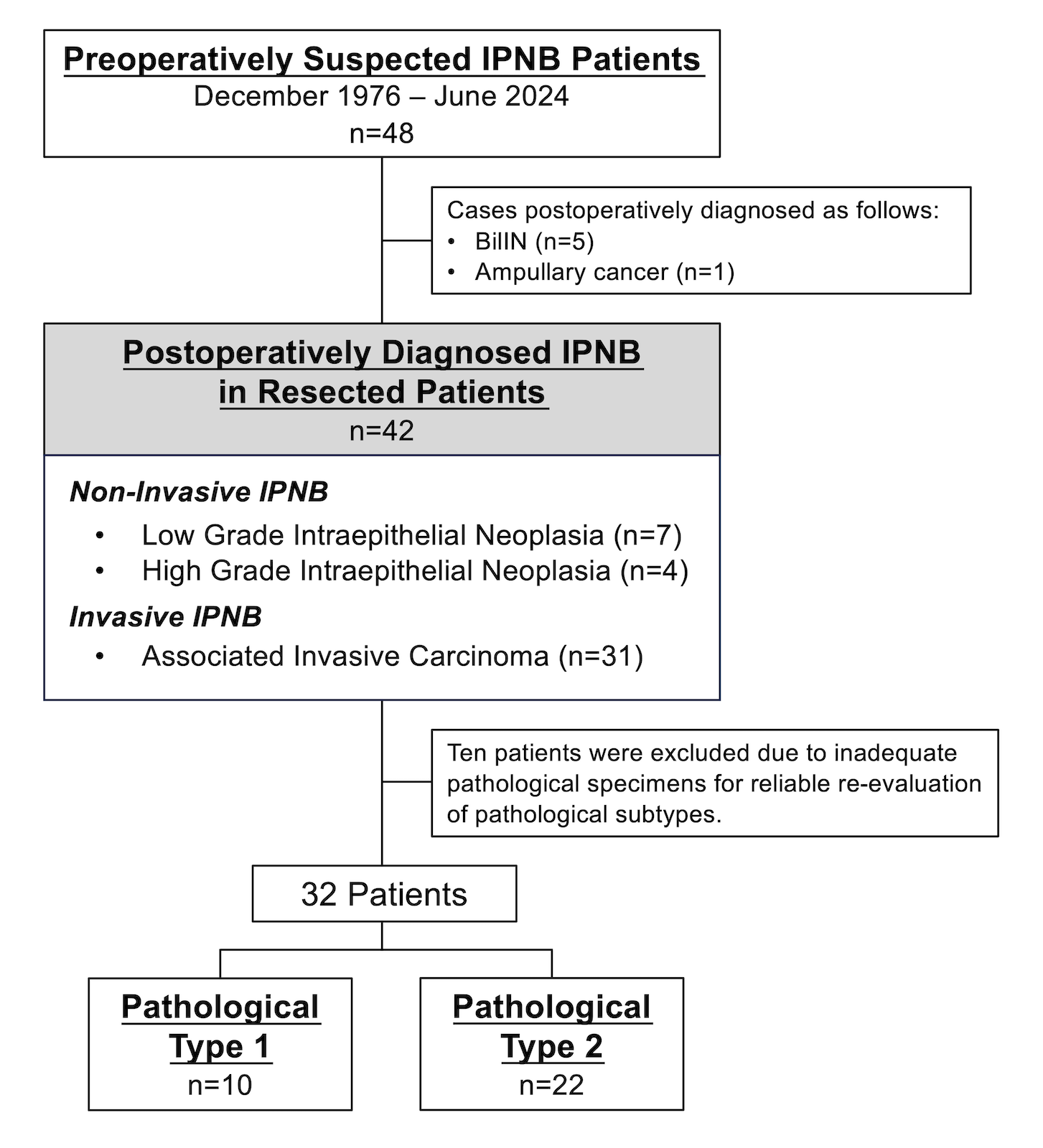

Supplement: S1 Fig — Abbreviation: BilIN, Biliary intraepithelial neoplasia; lPNB, intraductal papillary neoplasm of the bile duct. (TIFF) [file pone.0325081.s002.tiff]

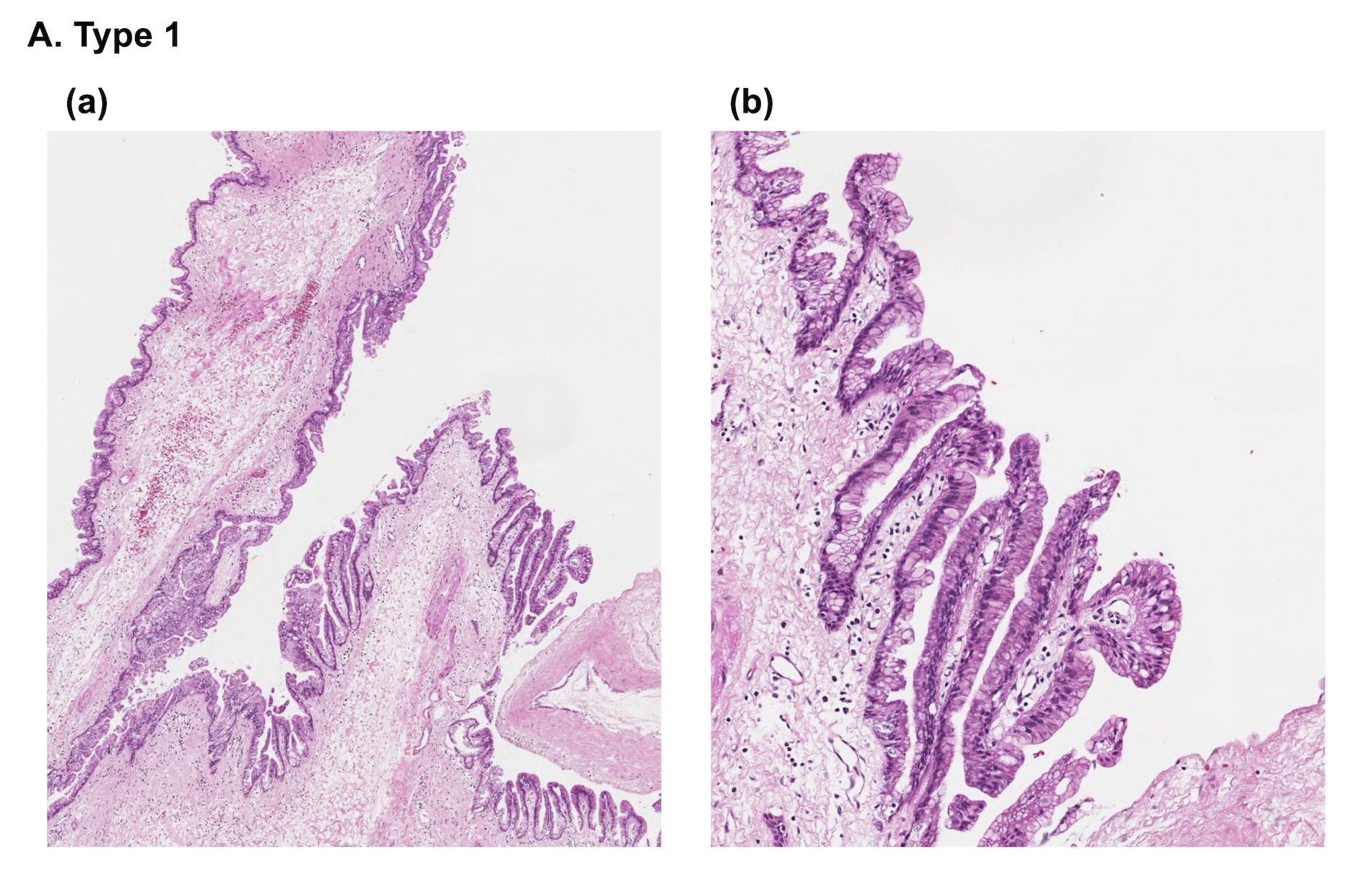

Supplement: S2 Fig — (A) Pathological Type 1 of IPNB. (A-a) A monotonous pattern of well-organized papillary-villous growth with a fine fibrovascular core appears within the cyst. Neoplastic cells show an overall well-organized arrangement. (A-b) A component of low to intermediate dysplasia is present. (B) Pathological Type 2 of IPNB. (B-a) In the dilated bile duct, various papillary architectures with thick fibrovascular stalks exhibit complex, irregular, and heterogeneous growth. (B-b) Neoplastic cells display nuclear hyperchromasia, stratification, and disordered polarity. Most cells show high-grade dysplasia. Abbreviation: lPNB, intraductal papillary neoplasm of the bile duct. (TIFF) (TIFF) [file pone.0325081.s003.tiff]

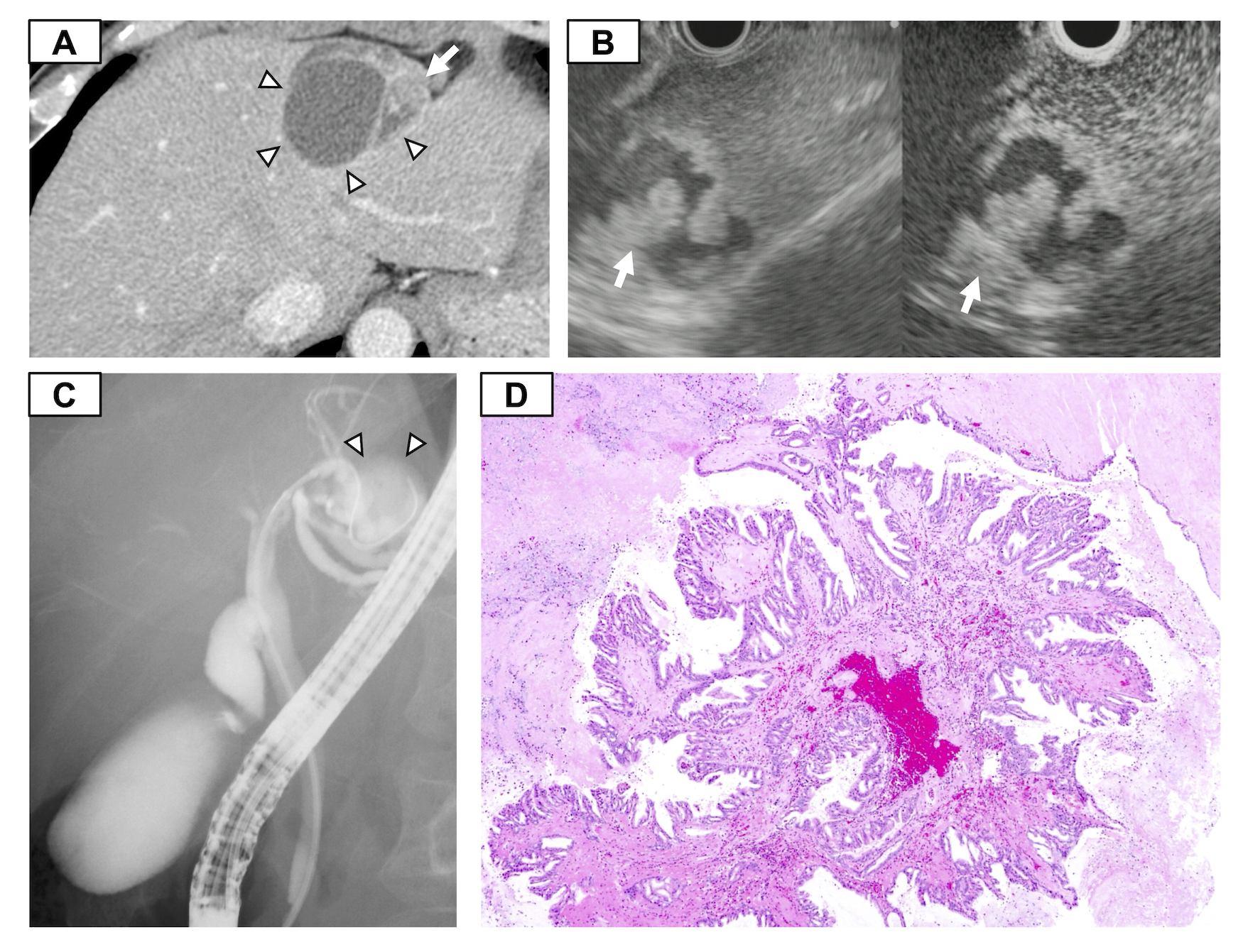

Supplement: S3 Fig — (A) CT imaging revealed a cystic lesion between liver segments 3 and 4 (arrowheads), with an enhanced mural nodule present within the cyst (arrow). (B) Endoscopic demonstrated mural nodules that were enhanced with a contrast agent (arrow). (C) ERC showed no bile duct dilation or extracystic lesions. (D) Histological examination identified a papillary growth tumor within the cystic lesion, leading to a diagnosis of IPNB associated with invasive carcinoma. Abbreviations: EUS, endoscopic retrograde cholangiography; IPNB, intraductal papillary neoplasm of the bile duct; US, ultrasound. (TIFF) [file pone.0325081.s004.tiff]

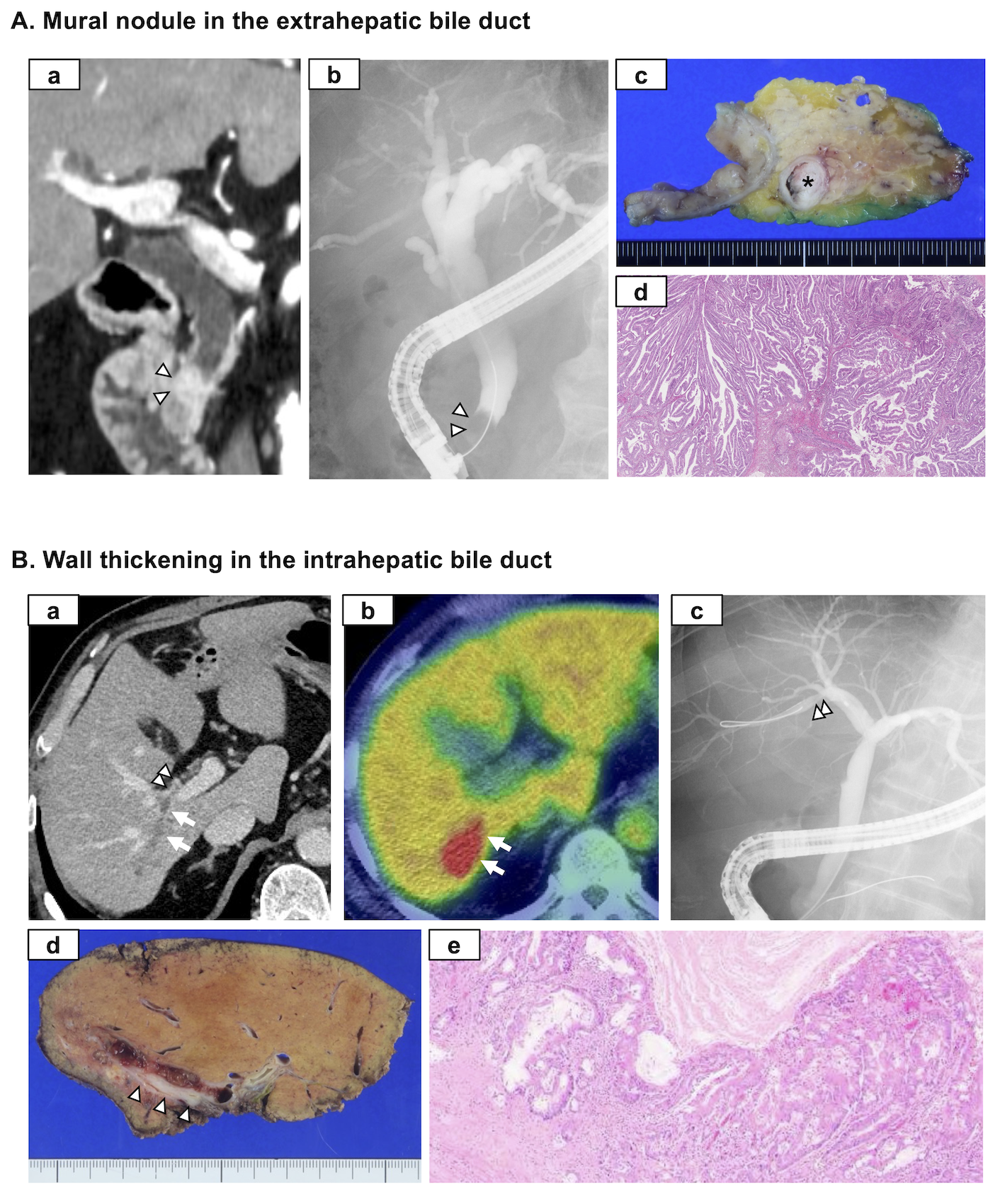

Supplement: S4 Fig — (A) A main duct-type IPNB with a mural nodule in the extrahepatic bile duct. (A-a) A mural nodule (arrowhead) was detected in the intrapancreatic bile duct on CT. (A-b) ERC demonstrated diffuse dilation of the entire bile duct proximal to the tumor. (A-c) The resected specimen obtained by pancreaticoduodenectomy showed a solid white tumor (*). (A-d) Histologically, the tumor exhibited a papillary architecture with heterogeneous growth patterns and was diagnosed as high-grade intraepithelial neoplasia (equivalent to CIS). (B) A main duct-type IPNB with localized wall thickening in the intrahepatic bile duct. (B-a) CT revealed wall thickening (arrowhead) and localized bile duct dilation (arrow) in the posterior branch. (B-b) PET-CT demonstrated FDG uptake in the lesion along the dilated posterior branch (arrow). (B-c) ERC showed wall thickening (arrowheads) in the corresponding area. (B-d) In the resected specimen, thickening of the posterior bile duct wall and a papillary tumor with mucin production were observed. (B-e) Histologically, the lesion consisted of papillary tumor cells, including invasive adenocarcinoma with heterogeneous growth patterns, leading to the diagnosis of IPNB with associated invasive carcinoma. Abbreviations: CIS, carcinoma in situ; CT, computed tomography; ERC, endoscopic retrograde cholangiography; FDG, fluorodeoxyglucose; PET, positron emission tomography; IPNB, intraductal papillary neoplasm of the bile duct. (TIFF) [file pone.0325081.s005.tiff]

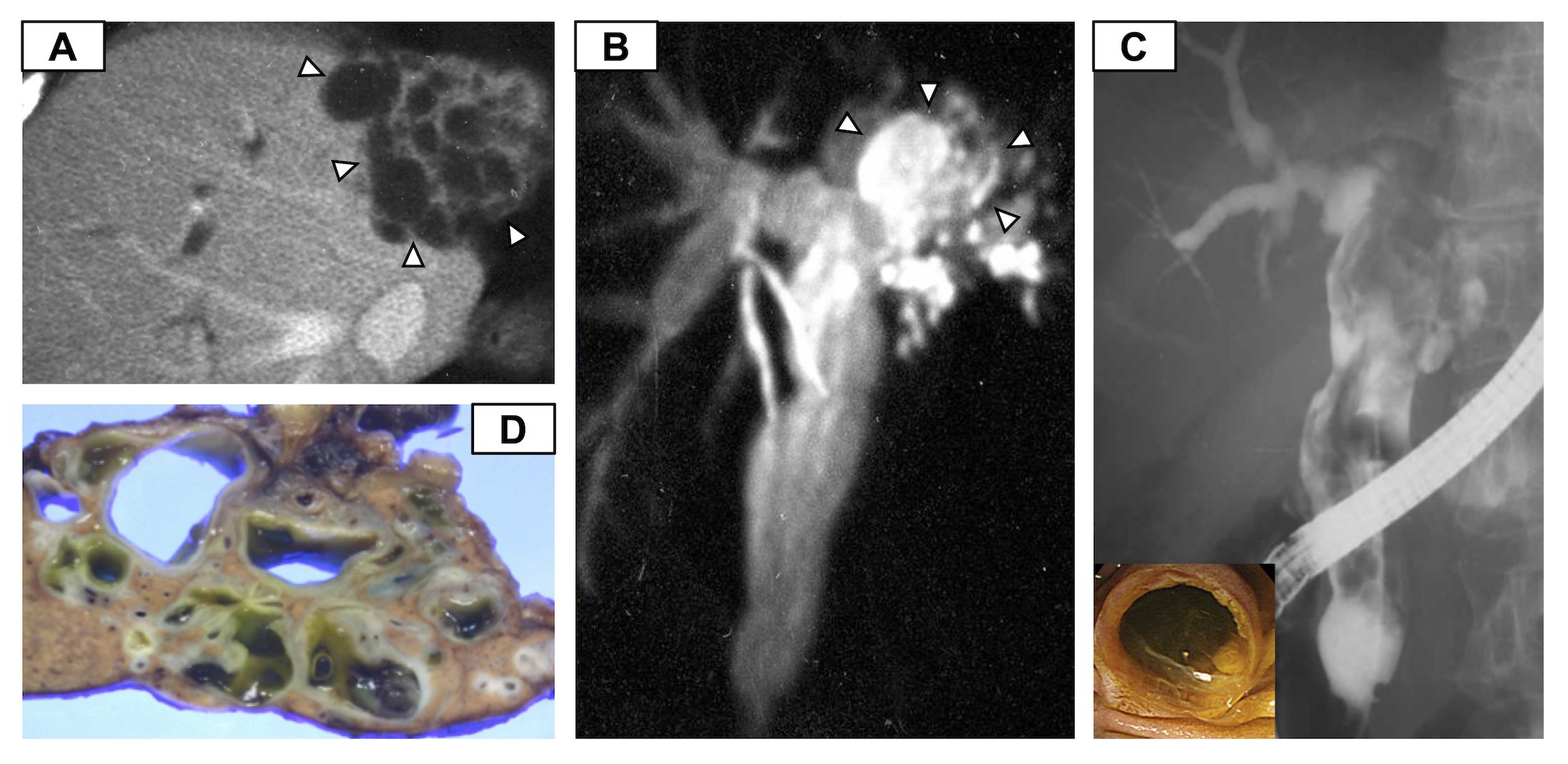

Supplement: S5 Fig — The mixed type IPNB is characterized by both cystic and bile duct features. (A) CT showed a multilobular cystic lesion (arrowheads) extensively occupying in the left lobe. (B) In MRCP, entire bile duct dilation without obstruction was identified besides the intrahepatic cystic lesion (arrowheads). (C) ERC showed defects in biliary truct and a bulging papilla of Vater, resulting from hypersecretion of mucin. (D) Resected specimen of the left lobe exhibited a multilobular cystic lesion with diagnosis of IPNB with associated invasive carcinoma. Abbreviations: CT, computed tomography; ERC, endoscopic retrograde cholangiography; MRCP, magnetic resonance cholangiopancreatography; IPNB, intraductal papillary neoplasm of the bile duct. (TIFF) [file pone.0325081.s006.tiff]

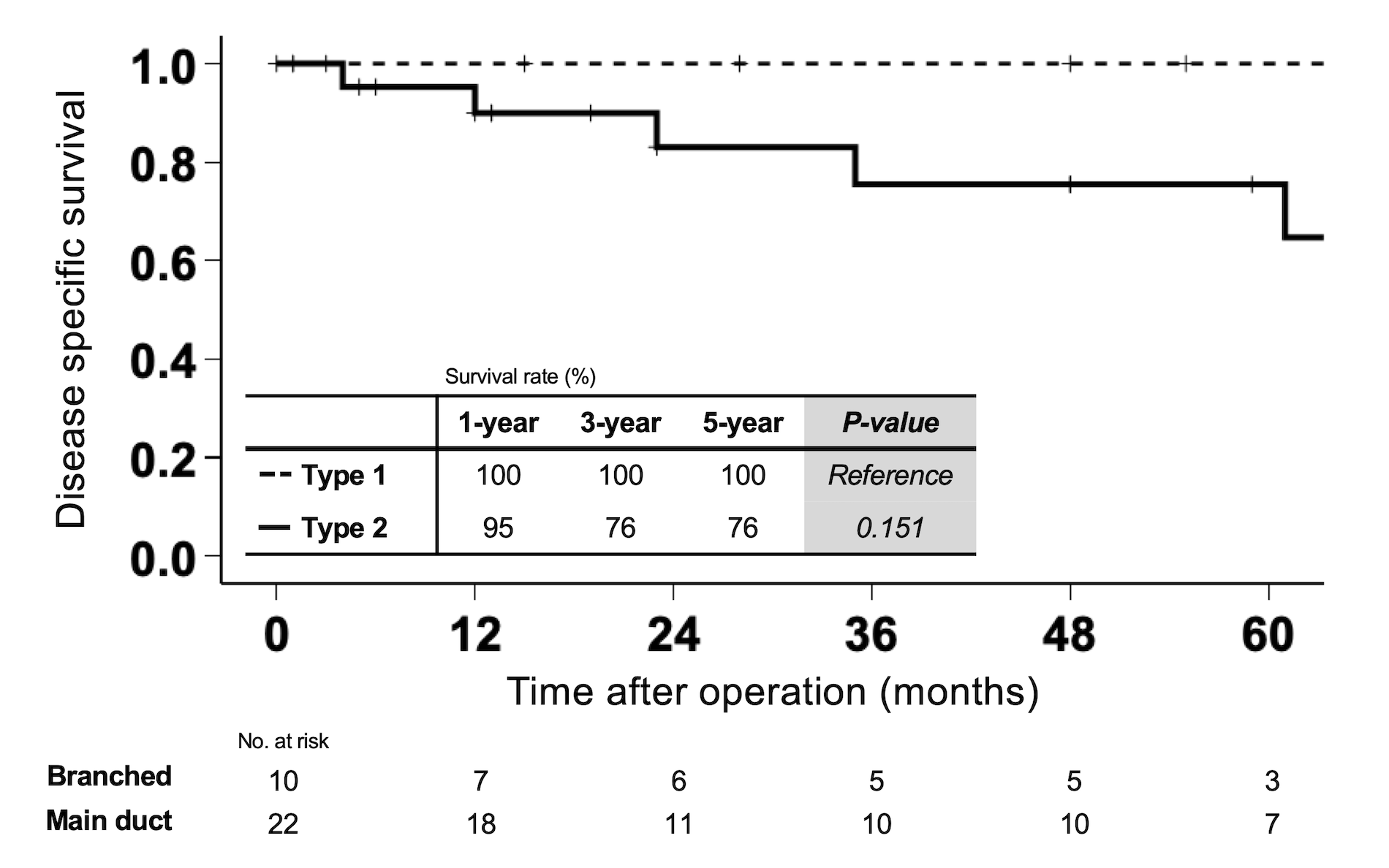

Supplement: S6 Fig — Postoperative DSS in patients with IPNB was compared according to pathological subtypes. Although the 1-, 3-, and 5-year survival rates in Type 2 (95%, 76%, and 76%, respectively) were lower than those in Type 1, the difference was not statistically significant (p = 0.151). Abbreviations: DSS, disease-specific survival; IPNB, intraductal papillary neoplasm of the bile duct. (TIFF) [file pone.0325081.s007.tiff]

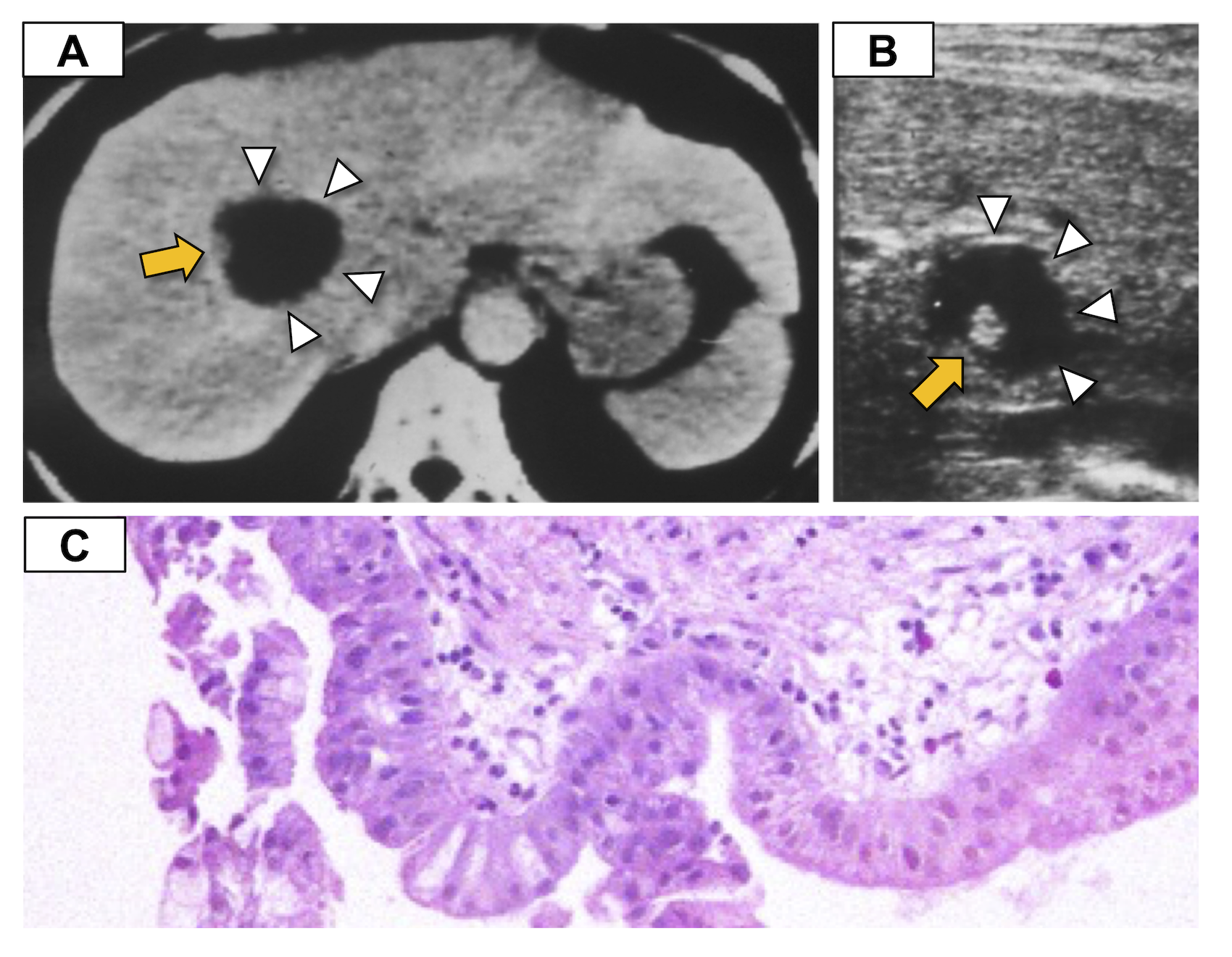

Supplement: S7 Fig — A 57-year-old female. (A) An intrahepatic cyst (arrowhead) was incidentally detected on CT. The lesion was in segments 5–8 and exhibited a mural nodule (yellow arrow). (B) Abdominal ultrasonography revealed an enhanced mural nodule (yellow arrow) within the cystic lesion (arrowhead), and a biopsy was performed on the nodule. (C) The biopsy specimen showed no evidence of malignancy. Since there were no abnormal features in the bile duct, such as dilation or wall thickening, she was diagnosed with branched-type IPNB. Although surgery was planned, the patient chose surveillance due to the absence of malignant findings. Abbreviations: IPNB, intraductal papillary neoplasm of the bile duct. (TIFF) [file pone.0325081.s008.tiff]

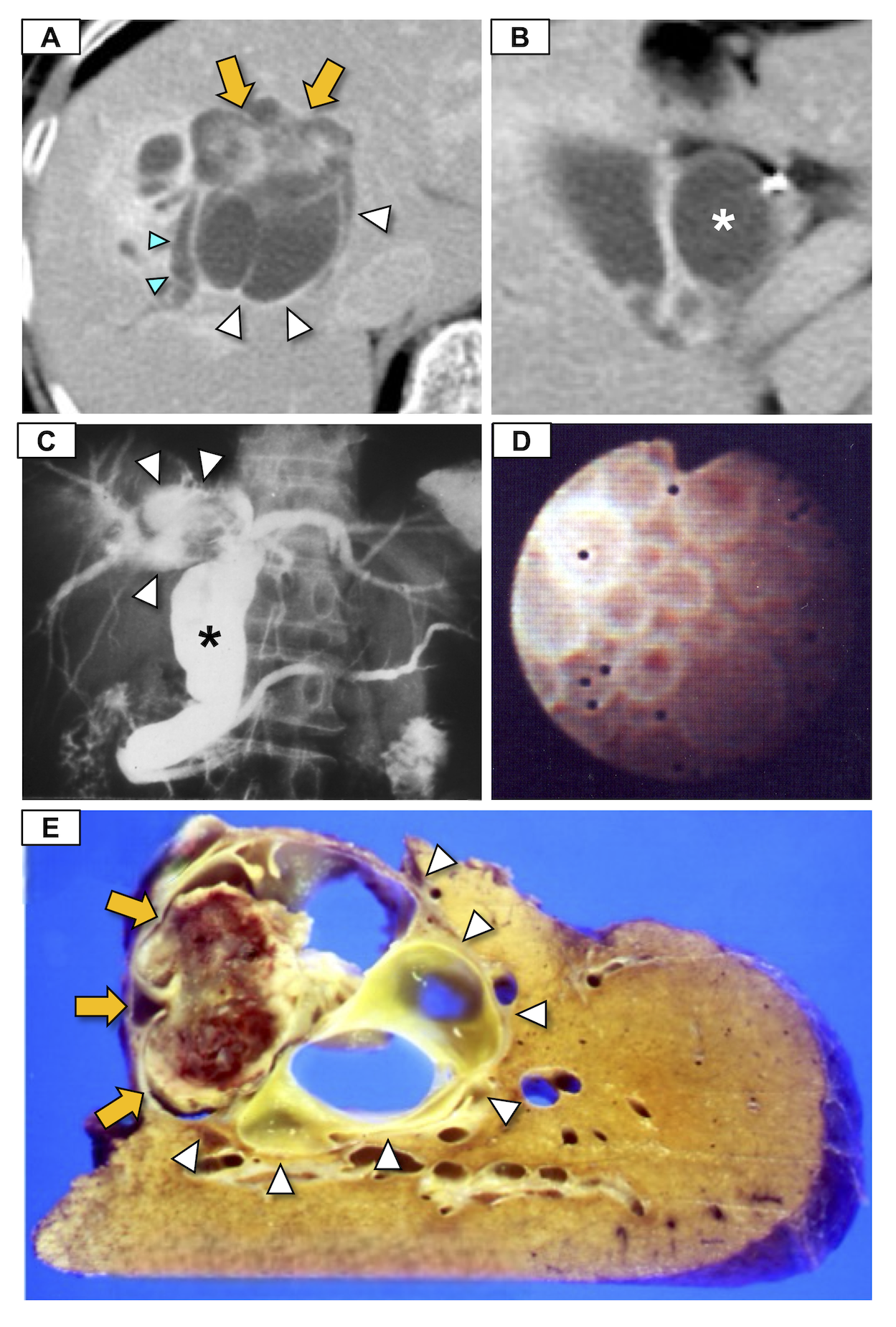

Supplement: S8 Fig — (A) (B) Fourteen years later, the cystic lesion (arrowhead) and the mural nodule (yellow arrow) had enlarged on CT. Additionally, dilatation of both the intrahepatic bile duct (blue arrowhead) and the extrahepatic bile duct (*) was observed. (C) Cholangiography revealed a cystic lesion (arrowhead) connected to the bile duct and dilated bile ducts without obstruction (*). (D) Cholangioscopy demonstrated mucin secretion and an intraluminally growing papillary tumor in the right main hepatic duct. Morphologically, the lesion was diagnosed as the mixed-type IPNB due to the presence of both the cystic lesion and bile duct dilatation without obstruction. Right hepatectomy and caudate lobectomy with extrahepatic bile duct resection were performed. (E) In the specimen, a mucin-producing papillary tumor (yellow arrow) was identified within the cystic lesion (arrowhead). While most of the cyst wall epithelium was adenoma, the tumor was an adenocarcinoma infiltrating the fibromuscular layer. Abbreviations: IPNB, intraductal papillary neoplasm of the bile duct. (TIFF) [file pone.0325081.s009.tiff]
