# Supplementary material for: A recent update on the morphological classification of intraductal papillary neoplasm of the bile duct: Correlation with postoperative prognosis and pathological features
Source: PLoS One. 2025 May 28;20(5):e0325081. doi: 10.1371/journal.pone.0325081 (PMC12118913; doi:10.1371/journal.pone.0325081)
Supplement: Attachment — Submitted filename: 2025.5.2_R1_Responses to Reviewers.docx [file pone.0325081.s013.docx]

**Dear Reviewers, Dr. Sean Bennett, and Dr. Emily Chenette,**

We sincerely appreciate your positive and constructive feedback on our manuscript entitled “**A Recent Update on the Morphological Classification of Intraductal Papillary Neoplasm of the Bile Duct: Correlation with Postoperative Prognosis and Pathological Features** **[ID: PONE-D-25-06283]**”. We have carefully addressed each comment and revised the manuscript accordingly. All textual changes in the manuscript, Figures and Tables are highlighted in blue.

We hope that the revisions meet your expectations, and that the manuscript is now suitable for publication. Please let us know if there are any further concerns or suggestions.

Once again, we deeply appreciate your careful consideration of our work.

Sincerely,

***Naohisa Kuriyama, M.D., Ph.D.***

Associate Professor

Department of Hepatobiliary Pancreatic and Transplant Surgery

Mie University Graduate School of Medicine

2-174 Edobashi, Tsu, Mie, Japan 514-8507

Phone: +81-59-232-1111

Mail: naokun@med.mie-u.ac.jp

***Reviewer #1***

**General Comment:**

The manuscript “A Recent Update on the Morphological Classification of Intraductal Papillary Neoplasm of the Bile Duct: Correlation with Postoperative Prognosis and Pathological Features” by Noguchi et al. examines 42 IPNBs and classifies them using a morphological system derived through radiologic characterization of IPNBs as branched type, main duct type, or mixed. They also classified them using the Japanese-Korean / WHO system as type 1 or type 2 based on histopathology. The authors evaluated postoperative survival based on several clinical-pathologic parameters, including by their proposed morphologic system. They found that mixed type IPNBs had worse survival compared to the others.

This paper represents an update on an earlier publication by this institution from 2013 and it includes more cases now. However, some fundamental flaws remain that make me skeptical of the validity of their proposed classification.

**Our response to the general comment:**

We sincerely appreciate your thoughtful and thorough evaluation of our manuscript. In this revised version, we have carefully addressed each of your comments and provided additional clarification to support the validity of our proposed morphological classification. We hope that our responses and revisions adequately address your concerns and improve the quality of the manuscript.

**Comment 1:** The mixed type only had 6 cases, a very small sample size that barely resulted in statistical differences on survival analysis. With so few cases, it is impossible to do meaningful multivariate regressions. My concern is that the mixed type may simply represent a more advanced stage disease, whereby tumor has expanded beyond its main duct or branch duct origin to involve adjacent regions. Although size or span can be difficult to measure in these types of lesions, I wonder if these are simply “larger” or at least more advanced stage tumors. In other words, I’m skeptical that the mixed morphology is an independent prognosticator that couldn’t be supplanted by other related, existing parameters such as size or stage.

**Comment 2:** There is some overlap between the intrahepatic versus extrahepatic anatomic classification as well as the type 1 and type 2 pathologic classifications with the proposed morphologic classification. Kim et al (reference 12) showed that intrahepatic versus extrahepatic location did not correlate with survival in a larger sample size. The authors argue that their morphologic system does correlate with survival due to more extensive spread in the mixed type. Couldn’t a similar classification be made for “mixed intrahepatic and extrahepatic involvement” instead? Do mixed intra and extra-hepatic tumours have different prognosis from those only involving intra or only involving extra?

**Our response to the comment 1 and 2:**

Your insightful comment highlighted a fundamental concern: the possibility that the poor prognosis observed in the mixed-type IPNB may simply reflect the adverse outcomes associated with larger or more extensively spreading tumors. If so, the need for a new morphological classification would be questionable. To address this concern appropriately, a comparison between our morphological classification and established indicators of tumor progression—such as tumor size or staging—would be required, ideally through multivariate analysis. However, as you also pointed out, the small sample size—the greatest limitation of our study—does not tolerate this statistical approach.

To address this issue despite the limited sample size, we conducted survival analyses comparing our morphological classification with indicators of tumor extent and progression. While indirect, we believe this clearly defined approach yields meaningful insights. First, we compared survival outcomes according to anatomical spread—limited to intrahepatic ducts, extrahepatic ducts, or involving both (Figure 2B). Survival did not significantly differ based on anatomical extent alone, suggesting that our morphological classification captures biological characteristics beyond tumor size or extent. Although the underlying factors remain unclear, the observed association with histopathological subtypes may be relevant. At the very least, morphological classification appears to be distinguishable from tumor extent. Second, to evaluate the impact of tumor progression, we focused on patients with invasive IPNB to enhance the validity of comparisons. Invasive IPNBs are treated as cholangiocarcinomas, and staging differs depending on tumor location—intrahepatic, perihilar, or distal bile duct—each with its own criteria. As you correctly noted, stage-based comparisons would be ideal, but due to differing staging systems, meaningful integration was not feasible. Instead, we used two common indicators of progression in bile duct cancers—depth of invasion and lymph node metastasis—to assess their association with prognosis. Both showed stronger statistical associations with prognosis than our morphological classification. However, these indicators are only available postoperatively, whereas our morphological classification can be assessed preoperatively, thus offering a distinct clinical advantage.

Taken together, our morphological classification may offer prognostic value beyond simply reflecting tumor size and its extent, and provide distinct clinical utility from tumor progression indicators by enabling preoperative prediction. The revisions are listed below.

Revisions to Figures:

- In **Figure 2**, DSS according to tumor extent was added. We also clarified that this analysis included all IPNB patients, distinguishing it from the survival analysis of patients with invasive IPNB shown in **Figure 3**. The legend for **Figure 2** has been revised accordingly.
- In **Figure 3**, to evaluate the impact of tumor progression, we focused on patients with invasive IPNB in survival analysis according to pathological tumor progression factors. The legend for **Figure 3** has been revised accordingly.

Revisions to manuscript:

- **On Page 13**: In the Results section “Postoperative survival by morphology and lesion extent in all IPNB cases”, we added the results of DSS by tumor extent as follows: As shown in **Figure 2B**, DSS did not show significant differences among the three groups based on tumor lesion extent (p=0.167 between intrahepatic and extrahepatic bile duct lesions, p=0.222 between intrahepatic and both lesions).
- **On Page 14:** In the Results section, we added a new subsection titled “Postoperative Survival by Pathological Tumor Progression Factors in IPNB with Associated Invasive Carcinoma,” which includes the following content: In thirty-one patients with IPNB with associated invasive carcinoma, DSS was compared based on pathological tumor progression factors. All patients with tumors confined to the mucosal (M) or fibromuscular (FM) layers survived without disease-specific death for 5 years after surgery. In contrast, patients with tumor invasion beyond the bile duct wall (serosal exposure [SE] or serosal invasion [SI]) had 1-, 3-, and 5-year DSS rates of 80%, 0%, and 0%, respectively, which were significantly lower (p < 0.001; **Figure 3A**). Patients with tumor extension to the subserosal (SS) or serosal (S) layers also had lower DSS compared to those with M or FM invasion (p = 0.037), although the difference became more evident after 5 years postoperatively. In patients with lymph node metastasis, the 1-, 3-, and 5-year DSS were significantly lower compared to those without lymph node metastasis. The DSS rates were 96%, 91%, and 91% versus 100%, 67%, and 67%, respectively (p=0.021, **Figure 3B**). In patients with residual tumor (R1 or R2), the 1-, 3-, and 5-year DSS were 86%, 64%, and 64%, respectively. Although these rates were lower than those in patients who underwent curative resection, the difference was not statistically significant (p=0.119, **Figure 3C**).
- **On Page 16, Line 288**: In the Discussion section, we emphasized that the morphological classification has significance beyond merely reflecting tumor extent, and that it offers clinical utility by being assessable preoperatively—unlike pathological factors that are strongly associated with prognosis but only available postoperatively. The revised sentences are as follows: However, lesion extent alone does not appear to fully account for the poor prognosis associated with the mixed type. As shown in Figure 2B, there were no significant differences in DSS among patients with intrahepatic, extrahepatic, or both intra- and extrahepatic tumor involvement. This suggests that our classification reflects not only the anatomical extent of the tumor but also underlying biological characteristics—for example, its association with pathological subtypes. We next examined the relationship between prognosis and cancer staging. In invasive IPNB, tumor stage is determined according to different classification systems based on tumor location—namely, intrahepatic, perihilar, or distal bile duct cancer—making direct comparison across cases impractical. Instead, we focused on two common oncologic factors: depth of invasion and lymph node metastasis, which can serve as surrogates for pathological staging. As shown in Figure 3, both were strongly associated with poor prognosis and, in terms of p-values, were even more predictive than morphological subtype. However, as these factors are only assessable postoperatively, our morphological classification offers a major advantage by enabling preoperative prognostic assessment and supporting treatment strategy.

**Comment 3:** The authors posit that the branch duct tumors are originating from peri-biliary glands, but since all the branch ducts tumors were intrahepatic, couldn’t they just be from smaller branches of intrahepatic ducts, rather than peri-biliary glands?

**Our response to the comment 3:** Thank you for your insightful comment. We revisited the literature regarding the histogenesis of IPNB and found that its origin is considered to be from both the peribiliary glands (PBGs) and the biliary epithelium (**Ref#1**). Since PBGs are distributed throughout both the intrahepatic and extrahepatic bile ducts, IPNBs originating from PBGs may potentially exhibit any of the morphological subtypes—branched, main duct, or mixed (**Ref#2, #3**). The same applies to lesions arising from the biliary epithelium. Therefore, although the precise cellular and tissue origins of IPNB remain to be unveiled, the idea that PBGs origin strictly corresponds to the branched type appears overly simplistic.

As you suggested, IPNBs derived from small bile ducts beyond the segmental ducts are likely to arise intrahepatically and to exhibit a branched-type morphology. We have added your consideration to the Discussion section and clarified that IPNB may arise from two potential origins as follows on **Page 15, Line 259**: Previous morphological classifications of IPNB considered the lesion location (intrahepatic vs. extrahepatic) as a key factor (**8,10,12**). In contrast, our new morphological criteria do not take lesion location into account. This is based on the understanding that IPNB can arise not only from biliary epithelial cells but also from peribiliary glands (PBGs) (**20**)—accessory glands distributed along both intrahepatic and extrahepatic bile ducts, which contribute to secretion and epithelial repair (**18,21**). Lesions derived from biliary epithelium, when arising in small bile ducts (after the segmental branches), typically may present as the branched type and be in the intrahepatic bile ducts. In contrast, PBG-derived lesions may occur irrespective of intrahepatic or extrahepatic bile duct, as PBGs are present throughout the biliary tract.

References:

#1 Nakagawa H, Hayata Y, Yamada T, Kawamura S, Suzuki N, Koike K. Peribiliary Glands as the Cellular Origin of Biliary Tract Cancer. Int J Mol Sci. 2018;19: 1745. doi:10.3390/ijms19061745

#2 Miyata T, Uesaka K, Nakanuma Y. Cystic and Papillary Neoplasm at the Hepatic Hilum Possibly Originating in the Peribiliary Glands. Case Rep Pathol. 2016;2016: 9130754. doi:10.1155/2016/9130754

#3 Uchida T, Yamamoto Y, Ito T, Okamura Y, Sugiura T, Uesaka K, et al. Cystic micropapillary neoplasm of peribiliary glands with concomitant perihilar cholangiocarcinoma. World J Gastroenterol. 2016;22: 2391–2397. doi:10.3748/wjg.v22.i7.2391)

**Comment 4:** With respect to survival analysis, disease specific survival is defined as per usual. However, the most common definition of “recurrence free survival” includes all deaths, but the authors chose to use a “recurrence-free disease-specific survival”, which is not a common oncologic outcome measure. I wonder if this very specific definition was chosen preferentially after multiple survival parameters were analyzed.

**Our response to the comment 4:** We sincerely apologize for the confusion caused. We would like to clarify that the recurrence-free survival (RFS) used in our analysis were not arbitrarily selected. Given that our study cohort includes patients treated as early as 1976, many patients have already died from non-disease-specific causes. Combined with the small sample size, we considered disease-specific mortality to be a more appropriate foundation for accurate time-to-event analysis related to tumor factors. Accordingly, we used disease-specific survival (DSS) as our primary survival endpoint and employed DSS + recurrence as the event definition for RFS.

However, as you rightly pointed out, this approach carries the risk of confusion, and since our intent was to assess recurrence alone, we decided to revise the analysis and simplify the content. Specifically, we now present cumulative recurrence rates instead of RFS. As also suggested by Reviewer #2, recurrence analysis should be limited to cases of invasive carcinoma. Therefore, we evaluated the relationship between morphological subtypes and cumulative recurrence rates in patients with IPNB with associated invasive carcinoma. The results have been added to **Figure 4** and described in the Results section on **Page 14, Line 242** as follows: Postoperative recurrence by morphology in IPNB with associated invasive carcinoma. The association between postoperative recurrence and morphological subtypes was evaluated in patients with invasive IPNB. No recurrence was observed in the branched type. In contrast, the mixed type showed a high recurrence incidence, with 1-, 3-, and 5-year cumulative recurrence rates of 25%, 25%, and 62%, respectively (p=0.074 vs. branched type, **Figure 4**). In the main duct type, the recurrence risk was intermediate, with 1-, 3-, and 5-year cumulative recurrence rates of 6%, 6%, and 13%, respectively, and no significant difference compared to the branched type (p=0.246).

**Comment 5:** Although the authors argue that the more aggressive nature of the mixed type could guide surgical management, the bulk of surgical decision making is still based on anatomical location, which dictates whether hepatectomy, bile duct resection, or pancreaticoduodenectomy is performed. In summary, although the finding that a mixed main/branched duct morphology potentially signals worse prognosis, this study has two main limitations. One is that the sample size for this category is very small (n=6). Secondly, the authors have not demonstrated that this morphology based system represents an independent prognosticator distinct from size or stage.

**Our response to the comment 5:** We sincerely appreciate your insightful and constructive feedback. As you rightly pointed out, the surgical approach for IPNB largely depends on the anatomical location of the lesion. In this context, we believe our proposed morphological classification provides clinical value in the determination of appropriate resection extent. For example, mixed-type IPNBs may require wider resections, while branched-type lesions could allow for more limited, minimally invasive approaches (laparoscopic or robotic). In cases where curative resection necessitates highly invasive procedures such as hepatopancreatoduodenectomy, this classification may support the rationale for surgical aggressiveness. In addition to these supportive roles, given the association between morphology and postoperative recurrence (**Figure 4**), our classification may contribute to postoperative treatment planning such as adjuvant therapy and follow-up strategies. Notably, because this classification can be assessed preoperatively, it may facilitate earlier and more individualized treatment strategies. These points have been incorporated into the revised Discussion section on **Page 17, Line 304**, as follows: We propose several practical implications of our morphological classification for surgeons. While the choice of surgical procedure (e.g., hepatic resection or pancreaticoduodenectomy) primarily depends on the anatomical location and extent of the tumor, our classification can aid in determining the appropriate resection extent. It serves as a rationale for surgical aggressiveness when considering highly invasive procedures such as hepatopancreatoduodenectomy. For example, mixed-type IPNB, associated with poor prognosis, may require wider surgical margins. In contrast, branched-type IPNB, which typically exhibits limited invasion and lower malignancy, may allow for more limited resection and is often amenable to minimally invasive approaches such as robotic or laparoscopic surgery. Postoperatively, the classification may also inform the need for adjuvant therapy and surveillance. The mixed type demonstrated a significantly higher recurrence rate, with over half of the patients experiencing recurrence within five years (**Figure 4**). In contrast, no recurrence was observed in the branched type, even when invasive components were present. These findings suggest that mixed-type IPNB may benefit from adjuvant therapy and intensive follow-up, while such measures might be unnecessary in the branched type. Crucially, this classification provides prognostic information preoperatively, supporting personalized treatment planning from the outset.

Furthermore, we fully acknowledge the two important limitations you noted: the small sample size and the lack of comparison with tumor progression indicators. We have addressed these issues as possible as we can (please refer to our responses to Comments 1 and 2), and have clearly stated these limitations in the revised Discussion section on **Page 18, Line 342** as follows: Limitations of this study include its retrospective, single-center design; however, the most critical limitation is the small cohort size. While we demonstrated an association between our morphological classification and patient prognosis, the small number of cases—particularly the limited number of mixed-type IPNBs with poor prognosis (n=6)—precludes definitive conclusions about its generalizability. For instance, to establish the classification as an independent prognostic factor, it would be necessary to compare it with known pathological risk factors such as depth of invasion and lymph node metastasis using multivariate analysis, which is not feasible given the current sample size. Nonetheless, we believe that the concept and criteria of our novel morphological classification are of significant value and hold considerable potential in the clinical setting. Future multicenter studies with larger cohorts are needed to objectively validate the significance and reliability of this classification system.

**We are deeply grateful for the considerable time and effort you dedicated to reviewing our manuscript.** We hope the revised manuscript addresses your comment.

**Thank you again for your invaluable assistance.**

Best regards,

Daisuke Noguchi

Naohisa Kuriyama

***Reviewer 2***

**General comment:**

Thank you for asking me to review this paper by Noguchi and colleagues. The authors have reviewed 42 cases of IPNB (32 cases available for path review). The have proposed a new morphological classification and examined its clinical impact. Overall, I found this paper to be interesting. The morphological classification is of value, although I suspect it will need a far larger study for this to be taken up into practice. It is very difficult to conclude much from a 42-patient study. I have made recommendations for improvement:

**Our response to the general comment:**

We sincerely appreciate your recognition of the value of our study. As you rightly pointed out, the limited number of cases remains the most significant limitation of this research. With future case accumulation and potential multi-institutional collaborations in mind, we conducted a thorough evaluation within the limitation of our current cohort to assess the clinical utility of our new morphologic classification. We would be grateful if you could also consider the originality and feasible concept of our proposed classification, in addition to the statistical outcomes.

**Comment 1:** How did you identify cases of IPNB? Was this from existing pathology records? From a database? By re-reviewing all existing cases of biliary tract tumors? How did you identify patients with IPNB prior to 2010, prior to the WHO definition being published? Did you review pathological specimens retrospectively? Did you search your institutional pathological database with keywords? This should be explained fully.

**Comment 2:** In the methods, it would be best not to repeat definitions, but rather to present methods by which you went about determining whether a patient had IPNB and its assigned WHO subtype. This is not described and certainly should be. More importantly, was this done retrospectively by re-reviewing all identified cases? How many pathologists re-reviewed the slides?

**Our response to the comment 1 and 2:** We appreciate your constructive feedback regarding the identification and diagnostic confirmation of IPNB cases. Below, we explain the study design in chronological order.

In 2010, the WHO 4th edition established the definition of IPNB that remains in use today. Cases diagnosed prior to its publication (before 2010) had already undergone pathological re-evaluation at the time of our previous study (published in 2013). Candidate cases were first extracted based on the diagnostic terms used at the time. Next, these pathological specimens were retrospectively reviewed and reclassified as IPNB according to the WHO 4th edition criteria. For cases diagnosed thereafter, pathological confirmation was made at the time of diagnosis using the latest WHO classification (either the 4th or 5th edition). Notably, the diagnostic criteria for IPNB have remained consistent between these two editions. Accordingly, all IPNB cases analyzed in this study had pathological diagnosis based on the same criteria.

In the current study, we additionally performed pathological re-evaluation to classify the pathological Type 1 and 2. Among the 42 IPNB cases, subtype assessment was possible in 32 cases; the remaining 10 cases were excluded from this analysis due to deterioration of pathological specimens. Importantly, the exclusion of these cases applied only to subtype evaluation and did not affect case inclusion for the primary analyses. All pathological evaluations were conducted by a single board-certified pathologist with over 25 years of experience.

Based on the above, the study design has been summarized in **S1 Figure**. Additionally, the Methods section "Definition of IPNB and Reevaluation of Pathological Subtypes 1 and 2" on **Page 7, Lines 109 and 125**, has been revised as follows:

- In this study, cases pathologically confirmed as IPNB according to the WHO classification were included. For cases diagnosed before the publication of the WHO 4th edition (2010) (**14**), pathological specimens had been retrospectively re-evaluated based on the WHO 4th edition criteria at the time of our previous study (published in 2013) (**8**) and confirmed as IPNB. Cases diagnosed after the previous study were pathologically confirmed at the time of diagnosis according to the latest WHO criteria (4th or 5th editions). Notably, the diagnostic criteria for IPNB did not substantially change between the 4th and 5th editions.
- The pathological subtypes, Type 1 and Type 2, were determined by additional pathological reevaluation based on the WHO 5th edition criteria (**1**). This reevaluation was performed by a single board-certified pathologist with over 25 years of experience.

**Comment 3:** Please remove all data from the methods. (e.g. paragraph presenting typical cases of sub-types of IPNB, with number of cases, this belongs in the results)

**Our response to the comment 3:** We sincerely appreciate your kind guidance. We apologize for deviating from the standard structure of a scientific manuscript. We have moved the description of the number of cases from the Methods section to the Results section. Please refer to the following revisions:

- **On Page 6, Line 104:** From December 1976 to July 2024 at our institution, IPNB patients who underwent surgical resection were analyzed the perioperative characteristics of IPNB, such as age, gender, symptoms, tumor findings, surgical procedure, histology, patient prognosis, and type of recurrence, according to our new classiﬁcation.
- **On Page 9, Line 173:** Among all 42 patients with IPNB, 10 (23%) were classified as the branched type, 26 (62%) as the main duct type, and 6 (15%) as the mixed type (**Table 1**).
- **On Page 13, Line 205:** 32 patients without evaluable pathological specimens for the pathological classification were divided into Type 1 (n=10) and Type 2 (n=22) (**S1 Figure**).

Additionally, representative cases have been compiled in the Supporting Information as **the S3, 4, and 5 Figures.** In the Methods section, we now describe only the criteria for morphological classification, and have provided a link to the representative cases at the end of the section. Please refer to the following revisions on **Page 8, Line 137**: IPNB patients were classified into three types—branched, main duct, and mixed—according to our newly proposed morphological criteria, which are based on a combination of cystic and bile duct imaging features (**Figure 1A**). **Figure 1B** illustrates each type.

Given the close embryological relationship between the biliary tract and the pancreas (**17**), IPNB can be morphologically classified in a manner analogous to pancreatic IPMN, into branched, main duct, and mixed types (**18**). Based on the international criteria for pancreatic IPMN (**19**), we first defined IPNBs with cystic lesions as the branched type, those with non-obstructive ductal dilatation as the main duct type, and those exhibiting both features as the mixed type. Based on these representative cases, cystic lesions were considered the “cystic factor,” a defining feature of the branched type, while bile duct dilatation without tumor obstruction was considered the “bile duct factor,” a defining feature of the main duct type. Bile duct wall thickening—sometimes accompanied by invasion into surrounding organs and mimicking cholangiocarcinoma (**11**)—was considered the feature to originate from large bile ducts, and thus, categorized as part of the bile duct factor. Classification criteria for the three morphological subtypes were then established based on the combination of these two factors. Representative clinical cases for each subtype are presented in **S3, 4, and 5 Figures**.

**Comment 4:** Please remove figure captions from the main manuscript, as it is very hard to read through properly.

**Our response to the comment 4:** We sincerely apologize for the confusion caused. While our original placement of figure legends followed the author’s guidelines, we agree with your comment that it may affect readability. In response, we have consolidated all figure legends into a single section at the end of the manuscript (on **Page 25**).

**Comment 5:** I would suggest removing p-values from table 1, as the number of patients is very small and the extreme number of hypothesis testing is not useful and subject to chance findings.

**Our response to the comment 5:** We appreciate your expert suggestion. We removed p-values from **Table 1**. For variables where statistical comparison is particularly important—namely, tumor lesion extent and the incidence of residual tumor among morphological subtypes—we have added footnotes (§, †) and included the corresponding p-values in the notes.

**Comment 6:** What drives the differences in postoperative oncologic outcomes between branch-, main-, and mixed-type lesions? Is it simply a matter of complete surgical resection? If so, there really is not much of a difference, other than mixed lesions being more diffuse and difficult to resect fully.

**Our response to the comment 6:** We are grateful for the reviewer’s valuable suggestions and kind guidance. To investigate whether factors beyond the difficulty of achieving curative resection—namely, the higher incidence of residual tumor—contribute to the poorer prognosis of the mixed-type IPNB, we conducted a survival analysis based on the anatomical extent of tumor spread, categorized as limited to the intrahepatic ducts, extrahepatic ducts, or involving both (**Figure 2**). If difficulty in achieving curative resection were the primary cause of poor prognosis, patients with tumors involving both intra- and extrahepatic bile ducts would be expected to have worse survival outcomes. However, our analysis found no significant association between anatomical tumor extent and prognosis.

The higher incidence of residual carcinoma in mixed-type IPNBs (**Table 1**) likely contributes, at least in part, to their poorer prognosis. On the other hand, the lack of prognostic relevance of tumor extent indicates that our morphological classification may reflect biological characteristics beyond simple tumor spread. While the underlying mechanisms remain unclear, the observed correlation between morphological and pathological subtypes may provide insights into their biological behavior.

The above revisions have been incorporated into **Figures 2** and its legend. The corresponding changes in the main text can be found in the revised sections indicated below.

- Results section on **Page 13, Line 222:** As shown in **Figure 2B**, DSS did not show significant differences among the three groups based on tumor lesion extent (p=0.167 between intrahepatic and extrahepatic bile duct lesions, p=0.222 between intrahepatic and both lesions).
- Discussion section on **Page 16, Line 289**: However, lesion extent alone does not appear to fully account for the poor prognosis associated with the mixed type. As shown in **Figure 2B**, there were no significant differences in DSS among patients with intrahepatic, extrahepatic, or both intra- and extrahepatic tumor involvement. This suggests that our classification reflects not only the anatomical extent of the tumor but also underlying biological characteristics—for example, its association with pathological subtypes.

**Comment 7:** Recurrence/oncologic outcomes should be limited to invasive cancer cases of course.

**Our response to the comment 7:** We are very grateful for your appropriate comments and valuable guidance. We fully agree with your suggestions. For tumor-related analyses, depth of invasion, lymph node metastasis, residual tumor, and cumulative recurrence rates were evaluated exclusively in cases with associated invasive carcinoma, as shown in **Figures 3 and 4**. The corresponding sections in the Results have also been revised as follows.

- Results section on Page 10, Line 189: Among IPNBs with associated invasive carcinoma, tumor invasion beyond the bile duct wall was observed in 0% of branched, 18% of main duct, and 25% of mixed types. In this population, the rate of residual tumor was significantly higher in the mixed type than in the branched type (75% vs. 0%, p=0.048). Lymph node metastasis was identified in four patients (18%), all of whom had main duct type IPNB.
- Results section on Page 14, Line 226: **Postoperative survival by pathological tumor progression factors in IPNB with associated invasive carcinoma.** In thirty-one patients with IPNB with associated invasive carcinoma, DSS was compared based on pathological tumor progression factors. All patients with tumors confined to the mucosal (M) or fibromuscular (FM) layers survived without disease-specific death for 5 years after surgery. In contrast, patients with tumor invasion beyond the bile duct wall (serosal exposure [SE] or serosal invasion [SI]) had 1-, 3-, and 5-year DSS rates of 80%, 0%, and 0%, respectively, which were significantly lower (p < 0.001; **Figure 3A**). Patients with tumor extension to the subserosal (SS) or serosal (S) layers also had lower DSS compared to those with M or FM invasion (p = 0.037), although the difference became more evident after 5 years postoperatively. In patients with lymph node metastasis, the 1-, 3-, and 5-year DSS were significantly lower compared to those without lymph node metastasis. The DSS rates were 96%, 91%, and 91% versus 100%, 67%, and 67%, respectively (p=0.021, **Figure 3B**). In patients with residual tumor (R1 or R2), the 1-, 3-, and 5-year DSS were 86%, 64%, and 64%, respectively. Although these rates were lower than those in patients who underwent curative resection, the difference was not statistically significant (p=0.119, **Figure 3C**).

In the tables, tumor superficial extension, along with depth of invasion, lymph node metastasis, and residual tumor, was re-analyzed exclusively in cases with associated invasive carcinoma; please refer to **Table 1** and **S1 Table**.

Furthermore, to accurately describe the presence or absence of invasive carcinoma in accordance with standard terminology, we unified the pathological terms throughout the manuscript as follows: low-grade intraepithelial neoplasm (corresponding to adenoma), high-grade intraepithelial neoplasm (corresponding to carcinoma in situ, CIS), and associated invasive carcinoma. These definitions have been clarified in the Methods section on **Page 7, Line 118**: The degree of dysplasia was classified as low-grade (including adenoma), high-grade (equivalent to carcinoma in situ, CIS), or invasive carcinoma (**13**).

**Comment 8:** It is not clear to me how surgeons should use this classification? Does this influence the decision to resect patients?

**Our response to the comment 8:** Thank you very much for your thoughtful and constructive feedback. We newly analyzed the association between postoperative recurrence and our morphological classification (**Figure 4)**, and based on this finding, we re-evaluated how surgeons may apply this classification in clinical practice.

In surgical decision-making, this classification may assist in determining the extent of resection. While the surgical procedure is primarily chosen based on the anatomical location and extent of the tumor, the mixed type, which is associated with poor prognosis and a high rate of residual carcinoma, may warrant more extensive resection. In contrast, the branched type often shows limited invasion and lower malignancy, supporting limited resection with minimally invasive approaches such as laparoscopic or robotic surgery.

Postoperatively, the recurrence risk stratified by morphology may inform decisions regarding adjuvant therapy and surveillance strategies. For instance, the mixed type shows a high recurrence rate and may require adjuvant treatment and intensive follow-up. In contrast, the branched type, even when associated with invasive components, might not require adjuvant therapy.

Importantly, our morphological classification allows for such prognostic assessments to be made before operation. This can support individualized treatment planning by influencing the timing of surgery, surgical indications, and the extent of resection based on anticipated prognosis.

The corresponding revisions are indicated below.

Revisions to Figures:

- In **Figure 4**, the cumulative recurrence rate according to morphological subtype was analyzed in patients with invasive IPNB. Its legend has been revised accordingly.

Revisions to manuscript:

- Results section on **Page 14, Line 243:** Postoperative recurrence by morphology in IPNB with associated invasive carcinoma. The association between postoperative recurrence and morphological subtypes was evaluated in patients with invasive IPNB. No recurrence was observed in the branched type. In contrast, the mixed type showed a high recurrence incidence, with 1-, 3-, and 5-year cumulative recurrence rates of 25%, 25%, and 62%, respectively (p=0.074 vs. branched type, **Figure 4**). In the main duct type, the recurrence risk was intermediate, with 1-, 3-, and 5-year cumulative recurrence rates of 6%, 6%, and 13%, respectively, and no significant difference compared to the branched type (p=0.246).
- Discussion section on **Page 17, Line 305:** We propose several practical implications of our morphological classification for surgeons. While the choice of surgical procedure (e.g., hepatic resection or pancreaticoduodenectomy) primarily depends on the anatomical location and extent of the tumor, our classification can aid in determining the appropriate resection extent. It serves as a rationale for surgical aggressiveness when considering highly invasive procedures such as hepatopancreatoduodenectomy. For example, mixed-type IPNB, associated with poor prognosis, may require wider surgical margins. In contrast, branched-type IPNB, which typically exhibits limited invasion and lower malignancy, may allow for more limited resection and is often amenable to minimally invasive approaches such as robotic or laparoscopic surgery. Postoperatively, the classification may also inform the need for adjuvant therapy and surveillance. The mixed type demonstrated a significantly higher recurrence rate, with over half of the patients experiencing recurrence within five years (**Figure 4**). In contrast, no recurrence was observed in the branched type, even when invasive components were present. These findings suggest that mixed-type IPNB may benefit from adjuvant therapy and intensive follow-up, while such measures might be unnecessary in the branched type. Crucially, this classification provides prognostic information preoperatively, supporting personalized treatment planning from the outset.

**Comment 9:** There are far too many figures for the sample size.

**Our response to the comment 9:** We sincerely appreciate your valuable suggestions. We have focused on the most important figures and moved the others to the Supporting Information.

**We honestly appreciate that you took the time and energy to help us improve the paper.** We hope the revised manuscript addresses your comment.

**Thank you again for your invaluable assistance.**

Best regards,

Daisuke Noguchi

Naohisa Kuriyama
